# Decompressive craniectomy of post-traumatic brain injury: an in silico modelling approach for intracranial hypertension management

**DOI:** 10.1038/s41598-020-75479-7

**Published:** 2020-10-29

**Authors:** Chryso Lambride, Nicolas Christodoulou, Anna Michail, Vasileios Vavourakis, Triantafyllos Stylianopoulos

**Affiliations:** 1 Department of Mechanical and Manufacturing Engineering, University of Cyprus, 1678 Nicosia, Cyprus; 2 Department of Medical Physics and Biomedical Engineering, University College London, London, WC1E 6BT UK

**Keywords:** Biomedical engineering, Computational models

## Abstract

Traumatic brain injury (TBI) causes brain edema that induces increased intracranial pressure and decreased cerebral perfusion. Decompressive craniectomy has been recommended as a surgical procedure for the management of swollen brain and intracranial hypertension. Proper location and size of a decompressive craniectomy, however, remain controversial and no clinical guidelines are available. Mathematical and computational (in silico) models can predict the optimum geometric conditions and provide insights for the brain mechanical response following a decompressive craniectomy. In this work, we present a finite element model of post-traumatic brain injury and decompressive craniectomy that incorporates a biphasic, nonlinear biomechanical model of the brain. A homogenous pressure is applied in the brain to represent the intracranial pressure loading caused by the tissue swelling and the models calculate the deformations and stresses in the brain as well as the herniated volume of the brain tissue that exits the skull following craniectomy. Simulations for different craniectomy geometries (unilateral, bifrontal and bifrontal with midline bar) and sizes are employed to identify optimal clinical conditions of decompressive craniectomy. The reported results for the herniated volume of the brain tissue as a function of the intracranial pressure loading under a specific geometry and size of craniectomy are exceptionally relevant for decompressive craniectomy planning.

## Introduction

Traumatic brain injury (TBI)—caused by an excessive force or penetrating injury to the head—is a major health concern worldwide, having short- and long-term adverse clinical outcomes, including death and disability^1,2^. TBI is defined as a head injury resulting from blunt or penetrating trauma or from an acceleration/deceleration force^1^, caused by a number of reasons, such as motor-vehicle crashes, falls, and assaults^2^. TBI can induce neurological damage owing to the primary injury (at the moment of impact) and the consequent secondary injury. The secondary brain injury is caused by brain swelling, leading to increased intracranial pressure (ICP) and subsequent decreased cerebral perfusion (ischaemia)^3^. Therefore, management of ICP in individuals is a crucial parameter in the treatment of TBI. The utilization of medications might not be able to reduce rising levels of ICP and, thus, a surgical procedure is often employed.

Decompressive craniectomy (DCC) is a useful surgical intervention for the management of brain injury and intracranial hypertension^1,4^, although the latter may be related to post-surgery adverse effects such as abnormalities in intracranial fluid movement^5^. DCC is a surgical procedure that involves removal of a portion of the skull, allowing the swollen brain to extend out of the cranium in order to relieve ICP. Normal ICP in adults ranges between 7 and 15 mm-Hg^6^, decompressive craniectomy is proposed when ICP levels exceed 20 mm-Hg for more than 30 min^7^. However, no guidelines are available for identifying the suitable location of a decompressive craniectomy or the effect of the size of the opening on the volume of the brain that can herniate through it. The size of the opening is directly related to the herniated volume of the brain tissue through the opening and, thus, the subsequent reduction of ICP. It plays also an important role during healing because large openings are associated with high rates of infection^8^. Therefore, for the optimal DCC decision and depending on the levels of ICP measured, pre-surgical estimation of the herniated volume of the brain, and how this would be impacted by the size of the opening (or the bone fragment), is of great clinical importance. Furthermore, craniectomy results in the development of mechanical stresses within the brain at the location of the opening, which should be also considered during the surgical procedure^8,9^. The most common locations of craniectomy are unilateral with an opening on the left or right hemisphere and bilateral with a bifrontal opening across both hemispheres. In addition, the optimal location for a craniectomy is thought to lie on the side of the swelling^8^; however, recent studies suggest that performing a craniectomy on the non-injured (opposite) side of the brain reduces tissue stresses^10^.

Computational models have been employed to investigate brain tissue biomechanics in response to high impact loading (e.g., see papers^11–13^) and to simulate brain deformations in the surgical setting (e.g., see papers^14,15^). However, efforts to simulate decompressive craniectomy have been very limited. In the early work of Gao et al. a biomechanical finite element (FE) model of DCC following traumatic brain injury was developed^16^. They simulated the brain deformation and ICP changes following unilateral fronto-parietal-temporal and bifrontal decompressive craniectomy, while they used their model to investigate potential ICP reduction with respect to trans-calvarial brain herniation minimization. In another study, Li and von Holst^10^ developed personalized FE models based on Computed Tomography (CT) images of six brain-injured patients. They developed a brain biomechanical model incorporating a poroelastic Saint Venant material with intrinsic viscoelastic behavior. The model was used to predict brain response on craniectomy, and it was validated with respect to brain surface movement before and after DCC. Fletcher and co-workers^9^ developed a hyperelastic (Ogden-based) model of an injured brain to simulate using ABAQUS different scenarios of DCC: unilateral, bilateral, bifrontal and bifrontal with a midline bar. Their study focused on correlating the craniectomy diameter, the herniated volume of the brain and the stresses developed at the boundary of the craniectomy. Their simulations highlighted that the location where brain injury occurs does not strongly impact the herniated volume predictions, while tissue volume suffering from shearing during DCC varied with respect to the craniectomy surface area. Similarly, Weickenmeier et al.^8^ modelled the head of an adult female individual using an isotropic hyperelastic FE approach and simulated progressive unilateral brain swelling following a circular opening in the left posterior skull. They investigated the sensitivity of skull opening relative to swelling site, where they have shown that small swelling volumes (28 to 56 ml) induce significant maximum principal strains (~ 30%) while they reported that stretch magnitude varies with opening site and the location where swelling develops.

Motivated by the previous studies, we propose here a FE model of brain injury that incorporates nonlinear biomechanics, describing the solid and fluid matter in the brain through a biphasic formulation, and the dynamics of ICP development due to swelling following a growth model. The aim of this contribution is to study the behavior of the swollen brain after DCC. To achieve this, different surgically pertinent DCC scenarios have been considered: unilateral craniectomy, bifrontal and a bifrontal with midline bar craniectomy. This allowed us to investigate the impact of the size and the location of the skull opening with respect to the ICP build up and the herniated volume of the brain tissue. To the best of our knowledge, the present study reports for the first time quantitatively how the herniated volume of the brain tissue relates to the pre-surgical intracranial pressure, and the spatial distribution of the stresses the brain experiences for the different DCC scenarios considered. Our results provide useful insights for better planning of DCC.

## Methods

### Three-dimensional reconstruction and model extraction from MR images

**Figure 1:**
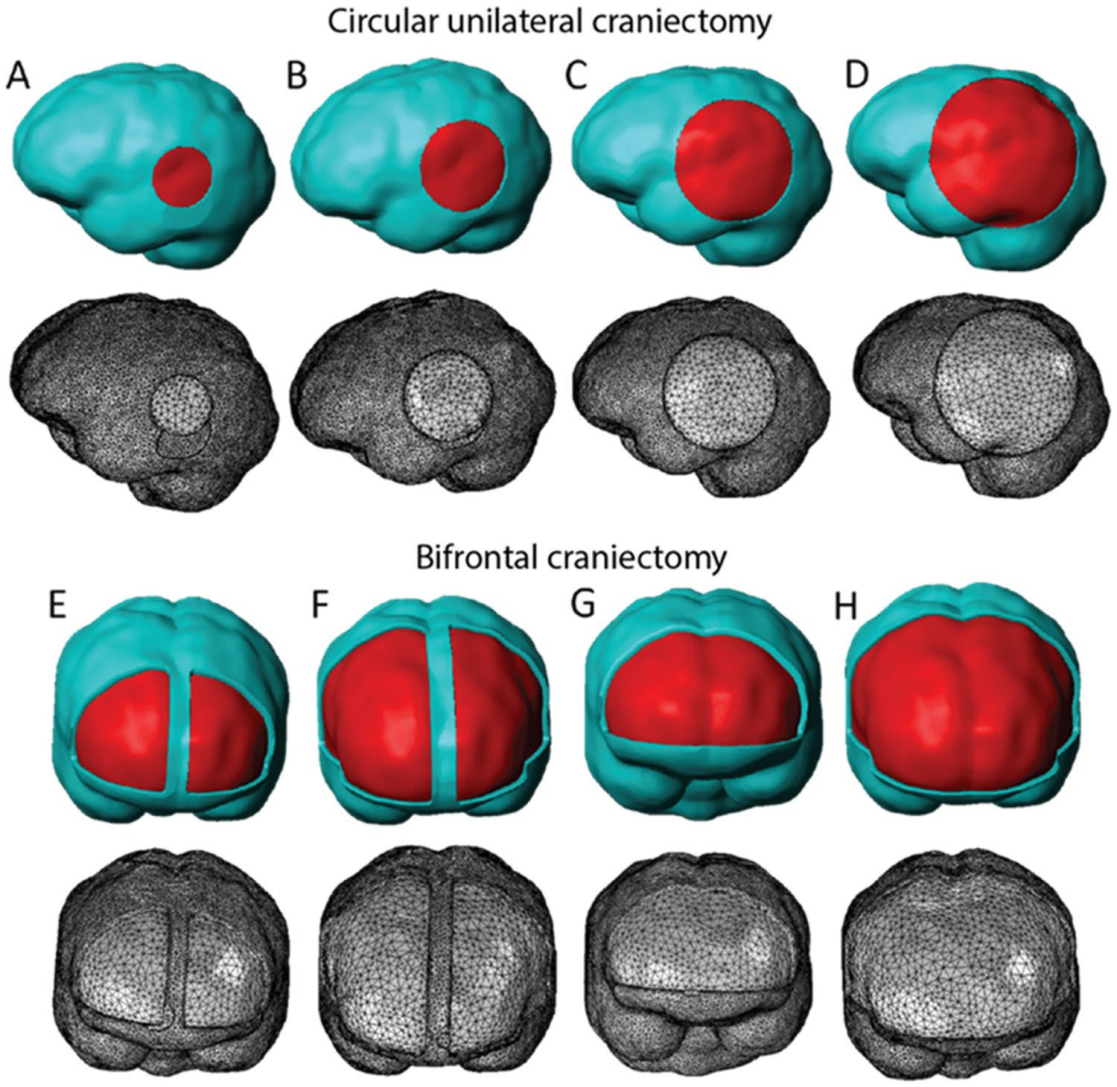
Three-dimensional finite element models of the different decompressive craniectomy scenarios considered. (**A**–**D**) Circular unilateral craniectomy models with cross-sectional area A = 1257 mm^2^ (r = 20 mm; entire model consisted of 164,926 tetrahedral FEs), A = 2827 mm^2^ (r = 30 mm; 162,566 tetrahedral FEs), A = 5027 mm^2^ (r = 40 mm; 155,829 tetrahedral FEs), and A = 7854 mm^2^ (r = 50 mm; 149,153 tetrahedral FEs), respectively. (**E**,**F**) Bifrontal with midline bar craniectomy models with total cross-sectional area of the craniectomy A = 6589 mm^2^ (157,098 tetrahedral FEs), and A = 15,826 mm^2^ (140,674 tetrahedral FEs), respectively. (**G**,**H**) Bifrontal craniectomy models with total cross-sectional area of the craniectomy A = 7344 mm^2^ (154,032 tetrahedral FEs), and A = 17,321 mm^2^ (135,284 tetrahedral FEs), respectively. Turquoise areas denote the internal surface of cranial base while the exposed brain surface is shown in red.

Magnetic resonance (MR) images of a healthy adult male were used to create an individualized, three-dimensional (3D) FE model of the skull and brain. The MR images were acquired from our previous study^17^ and were reused in this work to demonstrate in silico DCC biomechanics. The commercial software Simpleware ScanIP (version 6.0; Synopsys, Mountain View, USA) was used for the three-dimensional reconstruction and discretization of the brain geometry using volumetric FEs. Specifically, a mask was first generated from the images using Simpleware's “threshold” operation, which selects each pixel according to its brightness. Then, the “island removal” and “cavity fill” operations were used to eliminate small unconnected parts of the mask and fill any gaps on the model respectively. Smoothing was performed using the “Gaussian smoothing” operation. Subsequently, openings in the mask were created to represent unilateral and bilateral craniectomy on the FE model via the “3D editing” operation. The FE mesh was created and exported in a COMSOL-compatible mesh file. All craniectomy simulations were carried out using the commercial FE software COMSOL Multiphysics (version 5.2a; COMSOL Inc., Burlington, MA, USA). The masks and the meshes of the unilateral and bilateral simulations are depicted in Fig. 1. In all cases examined, the surface area of the opening was within a range of clinically relevance^9^.

### Biphasic formulation of the mathematical model

#### Mass conservation equations

Following the modelling approach of Gao & Ang^16^, brain biomechanics was simulated using a continuum-based biphasic model where, collectively, stromal and parenchymal tissue were described as a solid phase, while the interstitium was described as a fluid (continuum) phase. Thus, the conservation equations for the solid and the fluid phase read respectively:

1a $$ \frac{{d\varphi^{s} }}{dt} + \nabla \cdot \left( {{\mathbf{v}}^{s} \varphi^{s} } \right) = 0, $$

1b $$\frac{d{\varphi }^{f}}{dt}+\nabla \cdot \left({\mathbf{v}}^{f}{\varphi }^{f}\right)=Q,$$

where $\nabla$ is the gradient operator, ${\varphi }^{s}$ and ${\varphi }^{f}$ are the volume fractions of the solid and fluid phase respectively, so that: ${\varphi }^{s}+{\varphi }^{f}=1$*,* and ${\mathbf{v}}^{s}$ and ${\mathbf{v}}^{f}$ are the corresponding convection quantities for the solid and fluid phase as well. The solid velocity was numerically calculated as the time derivative of the displacement field of the solid phase, while the fluid velocity was evaluated using Darcy’s law, which describes flow through porous and fibrous media, via:

2 $$\left({{\varvec{v}}}^{f}-{{\varvec{v}}}^{s}\right) {\varphi }^{f}=-k \nabla {p}_{i,}$$

where *k* is the hydraulic conductivity of the tissue and *p*_*i*_ is the interstitial fluid pressure (IFP) of the brain tissue. IFP is the intracranial pressure (ICP) under normal conditions. In Eqs. (1a) and (1b), the source term $Q$ introduced the fluid exchange between the tissue and the vasculature, and it was modelled using Starling's approximation:

3 $$Q={L}_{p} \left(S/V\right)\left({p}_{v}-{p}_{i}\right)-{L}_{pl} \left({S}_{l}/{V}_{l}\right) \left({p}_{i}-{p}_{l}\right),$$

where ${p}_{v}$, ${L}_{p}$ and *S/V* are the micro-vascular pressure, hydraulic conductivity and vascular density of the blood vessels respectively, while ${p}_{l}$, ${L}_{pl}$ and *S*_*l*_*/V*_*l*_ are the corresponding quantities for the lymphatic vessels.

Summing up Eqs. (1a) and (1b), inserting Darcy’s Eq. (2) and taking the sum of the fluid and solid phase equal to unity, it reads:

4 $$-k {\nabla }^{2} {p}_{i}=Q-\nabla \cdot {{\varvec{v}}}^{s},$$

#### Linear momentum balance

The linear momentum equation can be written in the form of the total stress, ${{\varvec{\upsigma}}}_{\mathrm{tot}}$, of the two phases, the fluid phase, ${{\varvec{\upsigma}}}^{f}$, and the solid phase, ${{\varvec{\upsigma}}}^{s}$:

5 $$ \nabla \cdot {{\varvec{\upsigma}}}_{{{\rm{tot}}}} = 0\;{\rm{or}}\;\nabla \cdot \left( {{{\varvec{\upsigma}}}^{s} + {{\varvec{\upsigma}}}^{f} } \right) = 0. $$

The (Cauchy) stress tensor for the solid phase, ${{\varvec{\upsigma}}}^{s}$, was defined as: ${{\varvec{\upsigma}}}^{s}={{J}_{e}}^{-1}{{\varvec{F}}}_{e}\cdot (\partial \mathrm{W}/\partial {{{\varvec{F}}}_{e}}^{\mathrm{T}})$. Thus, the brain tissue was modeled as a homogeneous hyperelastic material according to previous pertinent studies^9,18,19^. A modified neo-Hookean strain energy density function was employed to describe brain’s mechanical behavior: $\mathrm{W}=0.5\left[\mu \left({I}_{1}-3\right)+K {\left({J}_{\mathrm{e}}-1\right)}^{2}\right]$, where for small deformations *μ* and *K* correspond to the shear and bulk modulus respectively, and ${I}_{1}$ is the first invariant of the deviatoric tensor of the elastic Cauchy-Green deformations, ${{\varvec{C}}}_{e}={{{\varvec{F}}}_{e}}^{T}\cdot {{\varvec{F}}}_{e}$, explained in the next paragraph. Respectively, the isochoric stress tensor for the fluid phase was defined through: ${{\varvec{\upsigma}}}^{f}={p}_{i}{\varvec{I}}$, with ***I*** being the identity tensor. However, it is very important to underline here that in view of the timescales involved during DCC interventions (typical range spans from minutes to hours), it is reasonable to ignore viscoelastic behavior of the brain biomechanics.

### Modeling brain tissue swelling following decompressive craniectomy

Brain swelling was modelled as a mass growth and deformation process inside the brain after TBI due to the internally applied and growth-induced stresses^20^. Therefore, brain swelling was described by the isotropic, inelastic deformation gradient tensor: ${{\varvec{F}}}_{g }={\lambda }_{g}\boldsymbol{ }{\varvec{I}}$, where ${\lambda }_{g}$ is the macroscopic (tissue) stretch ratio that represents isotropic brain tissue deformities. However, the multiplicative decomposition of the deformation gradient tensor, ${\varvec{F}}$, into its elastic and inelastic (growth) parts reads: ${\varvec{F}}={{\varvec{F}}}_{e}\cdot {{\varvec{F}}}_{g}$, where ${{\varvec{F}}}_{e}$ the deformation gradient tensor that describes the elastic (reversible) deformation related to mechanical interactions of the brain tissue. Therefore, from the definition of ${{\varvec{F}}}_{g}$, it is evident that the degree of swelling of the brain tissue is proportional to ${\lambda }_{g}$, which inherently would give rise to the increase in the intracranial pressure. Thus, in the present study, ${\lambda }_{g}$ serves as a modelling parameter as explained in the following paragraph describing the solution strategy of the proposed in silico method.

### Values of model parameters and boundary conditions

For simplicity and due to the small differences in the biomechanical properties reported for the gray and white matter tissue (e.g., see papers^9,17,21,22^) their biomechanical properties were assumed homogeneous throughout the entire 3D FE model. Thus, as in pertinent studies^17,22^, the shear and bulk modulus were set to 10 kPa and 30 kPa respectively, while the hydraulic conductivity, *k*, was taken equal to 6.7·10^–12^ m^2^(Pa s)^−1^. The vascular pressure, ${p}_{v}$, and permeability, ${L}_{p}$, was set to 4 kPa and 2.7·10^–12^ m^2^(Pa s)^−1^ respectively for the blood vessels, while for the lymphatic vessels was set to 0 kPa and 3.75·10^–4^ m^2^(Pa s)^−1^ respectively, whereas the vascular density was fixed to 7000 m^−1^ for both vessel types^17^.

Equations (4) and (5) of the decompressive craniectomy biomechanical model were discretized and solved numerically together using the commercial FE software COMSOL Multiphysics 5.2a (COMSOL, Inc., Burlington, MA, USA). The displacement (vector) field was discretized using quadratic Lagrange basis functions whereas the ICP (scalar) field was discretized using linear Lagrange basis functions, and the domain of analysis was meshed using tetrahedral FEs, as illustrated in Fig. 1. The skull was assumed as a non-deformable body and the skull/brain interface was fixed (i.e., u = 0) whereas brain tissue at the skull opening was unconstrained (i.e., traction-free boundary); therefore, brain tissue was permitted to deform due to swelling only through the opening. Also, a zero-flux boundary condition was applied for the fluid phase at the skull/brain interface and the fluid pressure variable at the skull opening was set to zero (${p}_{i}=0$; see Fig. 1 in Appendix B).

### Solution strategy

The proposed in silico approach for DCC modelling considers a two-step FE procedure: Firstly, a closed skull geometry FE simulation was carried out where ${\lambda }_{g}$ was increased from unity to 1.1 in order to explicitly model for the development of 10% swelling^8^. Assuming that the whole brain was considered injured, the brain swelling, ${\lambda }_{g}$, is prescribed on the entire brain of both hemispheres unless otherwise is noted. At this stage, the average ICP loading, ${p}_{i}$, was calculated numerically using COMSOL and was used as an input for the subsequent step of the simulation of the DCC procedure. Secondly, a DCC simulation was carried with an open skull (3D model), where the brain tissue was permitted to exert through the opening. Thus, the herniated volume is calculated as the difference between the brain volume before and after DCC. The tissue solid stresses and strains were calculated numerically using COMSOL at the post-process, for the different DCC scenarios (unilateral, bifrontal and bifrontal with midline bar) and for the ${\lambda }_{g}$ range of values specified in the first step.

## Results

### Unilateral decompressive craniectomy with circular cross-section

**Figure 2:**
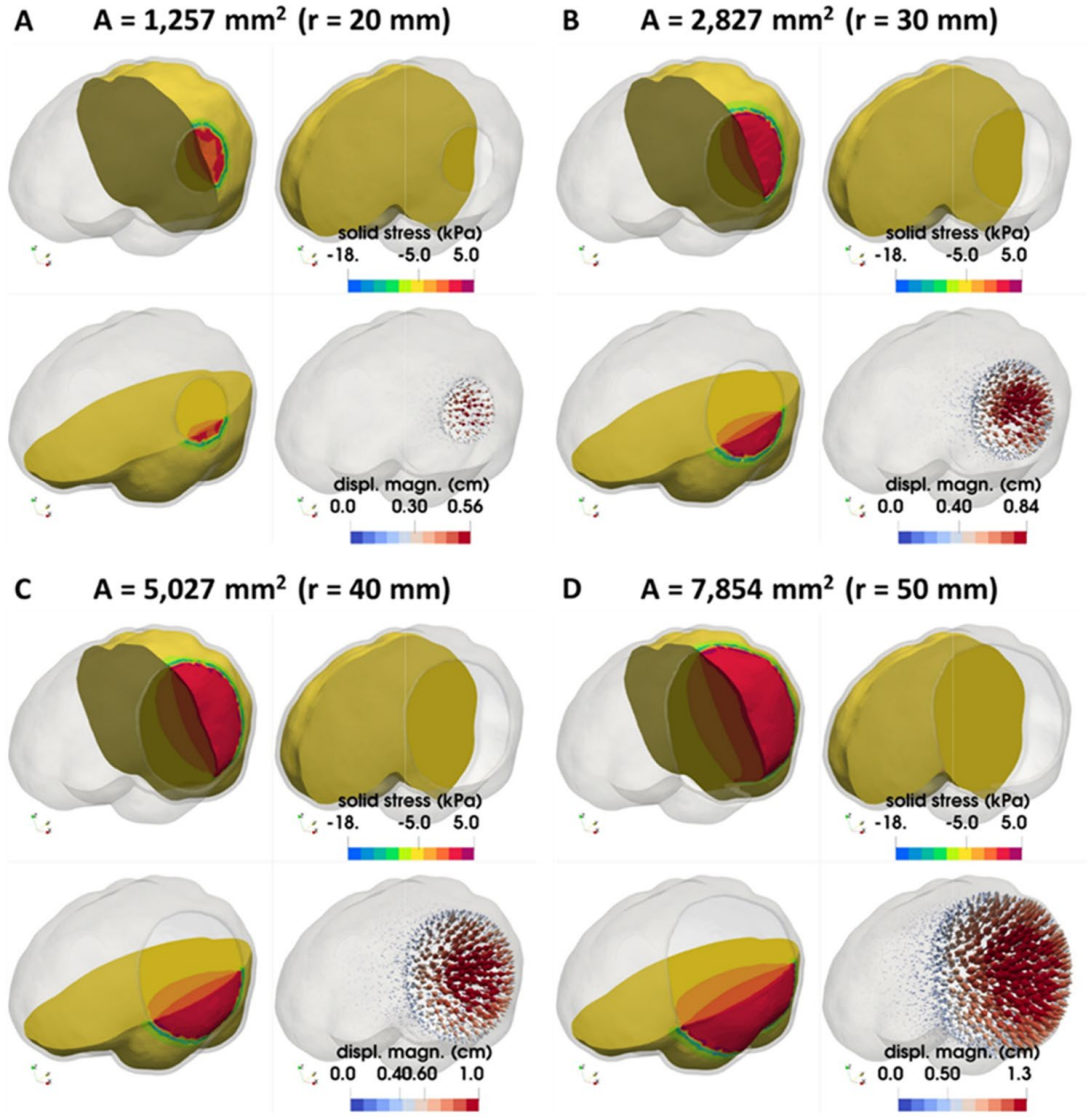
FE simulation results in a circular unilateral craniectomy. Dorsal, medial and caudal views of the brain model illustrating using contours the spatial distribution of brain tissue solid stress (in kPa) and using arrows the brain tissue displacement (in cm) for circular unilateral craniectomy and 10% brain tissue swelling (*λ*_*g*_ = 1.1 corresponding to ICP loading: 42 mm-Hg) and for a different skull opening radius: (**A**) 20 mm, (**B**) 30 mm, (**C**) 40 mm, and (**D**) 50 mm respectively.

**Figure 3:**
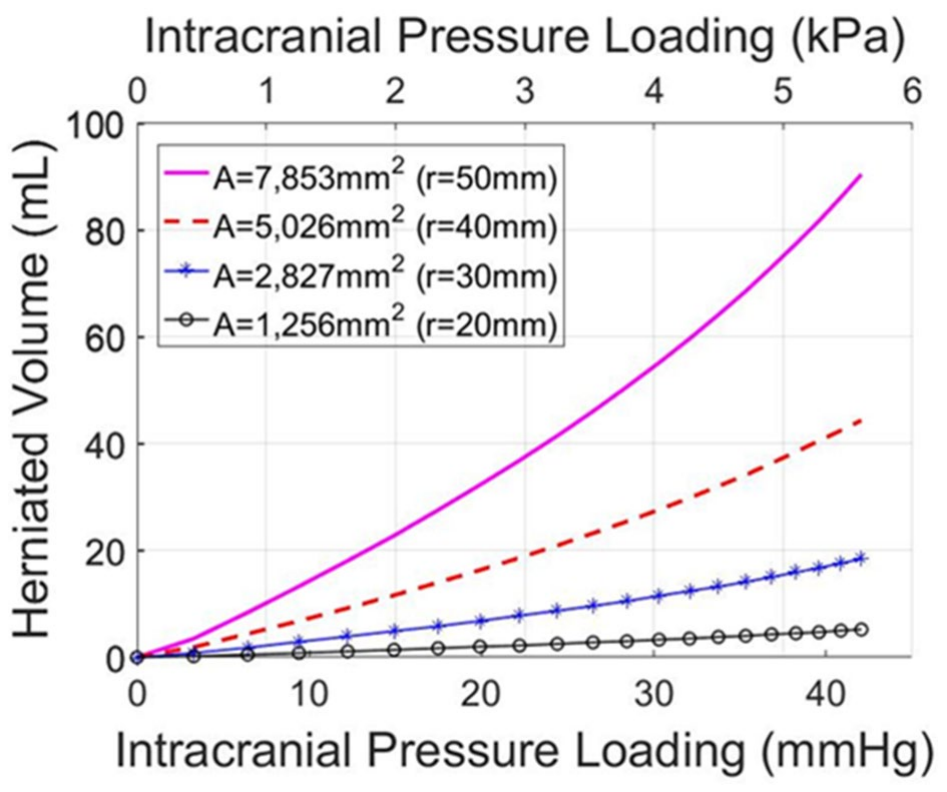
Brain herniated volume–ICP plots in a circular unilateral craniectomy. In silico predicted herniated volume of the brain tissue following craniectomy for different circular openings as a function of the intracranial pressure loading prior to DCC.

We first set out to investigate the effect of the size of the craniectomy on the developed stresses and deformations of the brain as well as on the herniated volume of the brain tissue that goes out of the skull during craniectomy due to tissue swelling after TBI. The radius of the circular opening varied from r = 20 mm to r = 50 mm (Fig. 1). As the size of the craniectomy opening increases, the total displacement of the brain tissue near the opening increases (Fig. 2). The magnitude of the stresses is not significantly affected by the size of the craniectomy (see also plots of max. stress versus ICP in Fig. 2A in Appendix B). The maximum compressive stress value for a 10% swelling (i.e., ${\lambda }_{g}=1.1$) is 18 kPa for radius of craniectomy 30 mm (Fig. 2). The line plots in Fig. 3 present the ICP prior to the decompressive craniectomy procedure, in relation to the predicted by the model outgoing volume following craniectomy for the different circular openings considered. Clinically measured values of intracranial pressure elevation caused by TBI are reported in^23^ within the range of 18–80 mm-Hg (2.4–10.7 kPa). However, the proposed model predicts the herniated volume of the brain tissue to range from 20 to 90 ml for the two largest openings, which falls within the range measured in the clinical study^24^.

### Bifrontal and bifrontal with midline bar decompressive craniectomy

**Figure 4:**
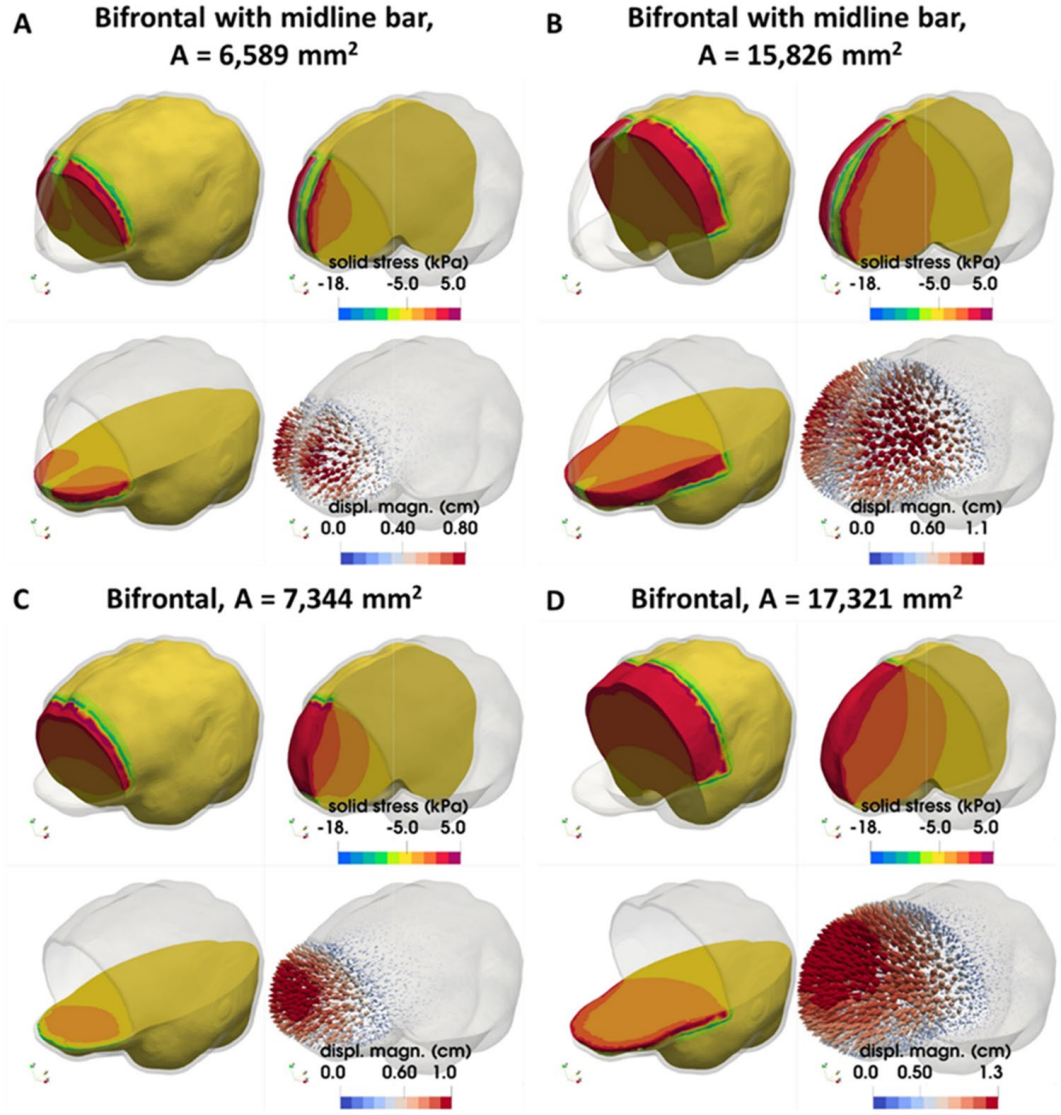
FE simulation results in a bifrontal craniectomy (with or without midline bar). Spatial distribution of brain tissue solid stress (in kPa) and displacement (in cm) for a (**A**,**B**) bifrontal with midline bar craniectomy and (**C**,**D**) bifrontal craniectomy and for 10% brain tissue swelling (*λ*_*g*_ = 1.1 corresponding to ICP loading: 42 mm-Hg).

**Figure 5:**
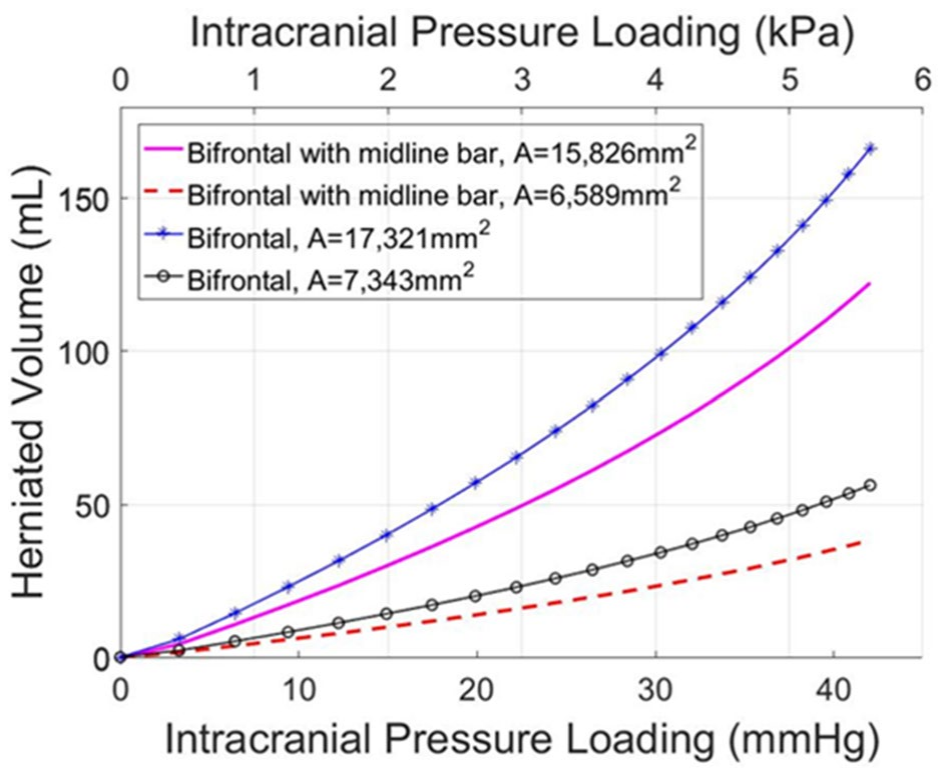
Brain herniated volume–ICP plots in a bifrontal craniectomy (with or without midline bar). In silico predicted herniated volume of the brain tissue following craniectomy for different bifrontal craniectomy openings as a function of intracranial pressure loading prior to DCC.

Subsequently, we incorporated two common craniectomy scenarios, different to the above subsections: the bifrontal and the bifrontal with a midline bar. As shown in Fig. 1, the considered opening areas for bifrontal with midline bar craniectomy were 6589 mm^2^ and 15,826 mm^2^, while for the bifrontal craniectomy scenario the opening areas were 7344 mm^2^ and 17,321 mm^2^, respectively. Thus, considering two decompressive craniectomy scenarios and for two opening areas in each case, we investigated the relationship between ICP and herniated volume, and therefore studied how bifrontal craniectomy simulation results compare to the unilateral DCCs above. The total displacement and stresses of the brain tissue are shown in Fig. 4. An increase in total displacement of brain tissue is observed as the size of the opening increases. In the cases of bifrontal, the total displacement of brain tissue is larger than in cases of bifrontal with midline bar. The maximum compressive stresses are found near the ends of the opening for bifrontal with midline bar craniectomy of 15,826 mm^2^, reaching 21.1 kPa for 10% swelling (see also plots of max. stress versus ICP in Fig. 2B in Appendix B). The distribution of stresses in the brain tissue varies for each geometry. The intracranial pressure prior to the decompressive craniectomy procedure in relation to the predicted herniated volume following craniectomy and for the different openings employed are shown in Fig. 5. Bifrontal openings can result in significantly larger herniated volumes compared to unilateral openings, whereas changes in the stress levels (maximal values) are observed here to be marginal—the mechanical stress distribution pattern drastically changes amongst the DCC scenarios considered.

### Decompressive craniectomy at injured and non-injured side and midline swift

**Figure 6:**
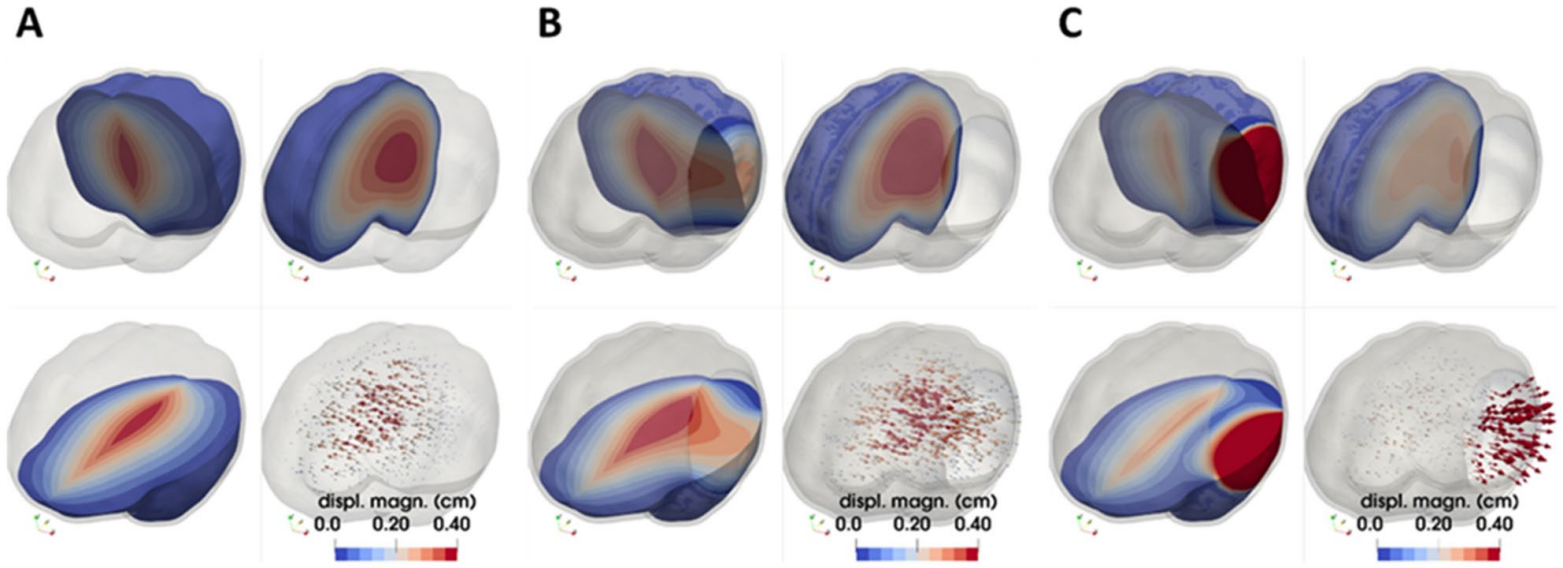
FE simulation results of brain tissue displacements before and after a unilateral craniectomy—dependence with respect to the injury site. Resultant displacement of brain tissue (in cm) for three different cases of a single hemisphere (half-brain) injury: (**A**) pre-surgical case (no skull opening); (**B**) DCC opening opposite side to the injured brain hemisphere and (**C**) opening on the same side with the injured hemisphere. The radius of the opening in (**B**) and (**C**) was r = 40 mm.

Next, we interrogated the potential effect applying DCC either at the same side of the skull where tissue injury has occurred or to the opposite side of the skull, and study how this affects the shifting of the brain midline. Using the same MR data, three different models were prepared: The first FE model considered an injured brain and a closed skull to represent the situation prior to craniectomy, where only one hemisphere of the brain was considered injured (swollen). This model was used as a “control” to estimate the maximum midline shift of the brain, which was evaluated by calculating the maximum displacement (with respect to the sagittal plane) of the cerebral falx. The other two FE models considered an injured brain with a skull with unilateral craniectomy either at the same or at the opposite hemisphere with respect to the injury site. The displacement of brain tissue for the three different models was calculated, as shown in Fig. 6. The midline shift of the closed skull for 10% swelling (${\lambda }_{g}=1.1$) was 4.1 mm. When the injury and the opening were on the opposite side, the midline shift was 4.6 mm (12% larger with respect to the control) because the injured side of the brain expanded against the non-injured side, whereas when the injury and the opening were on the same side of the brain, the midline shift was 3 mm (27% approximately smaller with respect to the control). This is an intuitive result because the preferred path of tissue deformation, and therefore pressure decompression, was towards the opening of the skull.

### Brain tissue biomechanical properties affect herniated volume

**Figure 7:**
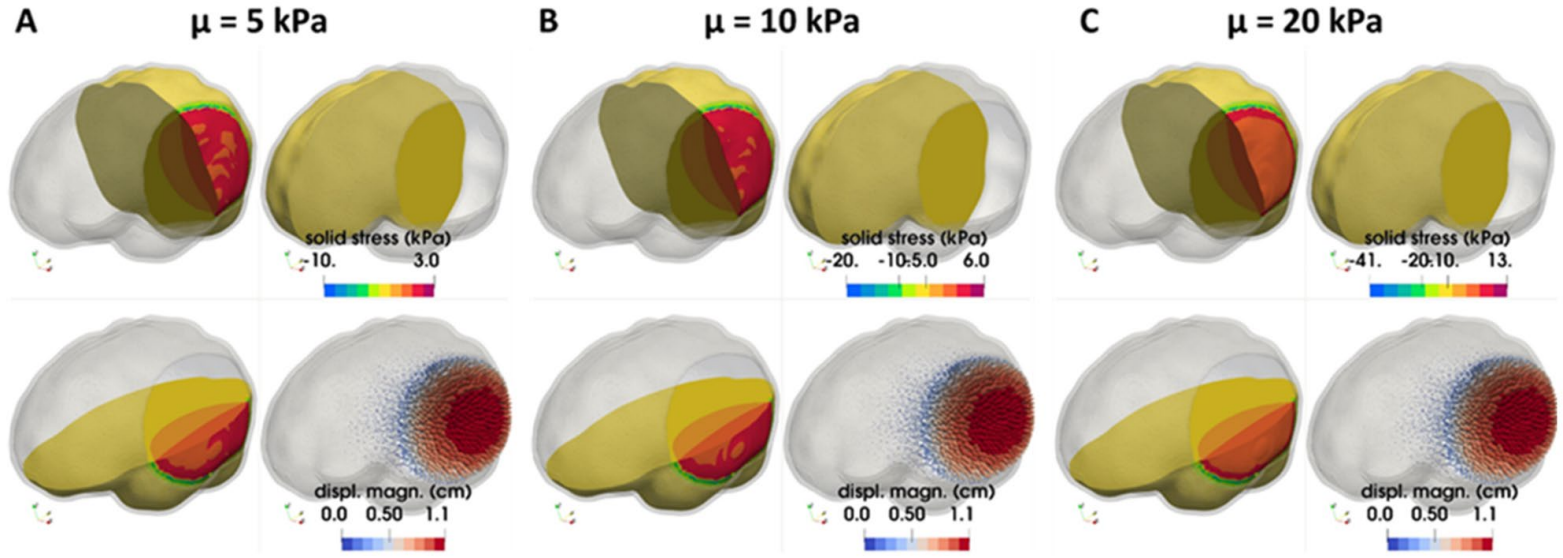
FE simulation results in a circular unilateral craniectomy—dependence with respect to shear modulus, μ. Dorsal, medial and caudal views of the brain model illustrating using contours the spatial distribution of brain tissue solid stress (in kPa) and using arrows the tissue displacement (in cm) for a circular unilateral craniectomy, 10% brain tissue swelling (*λ*_*g*_ = 1.1) and for different values of the shear modulus respectively. The radius of the opening was kept r = 40 mm.

Finally, the sensitivity of the brain behavior following craniectomy with respect to its tissue biomechanical properties was investigated. The shear modulus of the brain tissue varied within physiological values from 5 to 20 kPa^25,26^ while the bulk modulus was fixed, and simulations were repeated for the unilateral craniectomy scenario with a 40 mm radius opening. Figure 7 shows contours of the solid stress developing in the brain after DCC (in dorsal, medial and caudal view) and the displacement vector field shown using arrows (colored with respect to their magnitude). As seen from this figure, brain tissue displacement is not significantly affected by the value of the shear modulus, with the max displacement being approximately 10.5 mm, during the decompressive craniectomy procedure. The solid stresses, however, varies significantly and the maximum compressive solid stresses reached values of 8.9, 17.9, and 35.9 kPa for shear modulus values of 5, 10, and 20 kPa respectively (Fig. 7).

**Figure 8:**
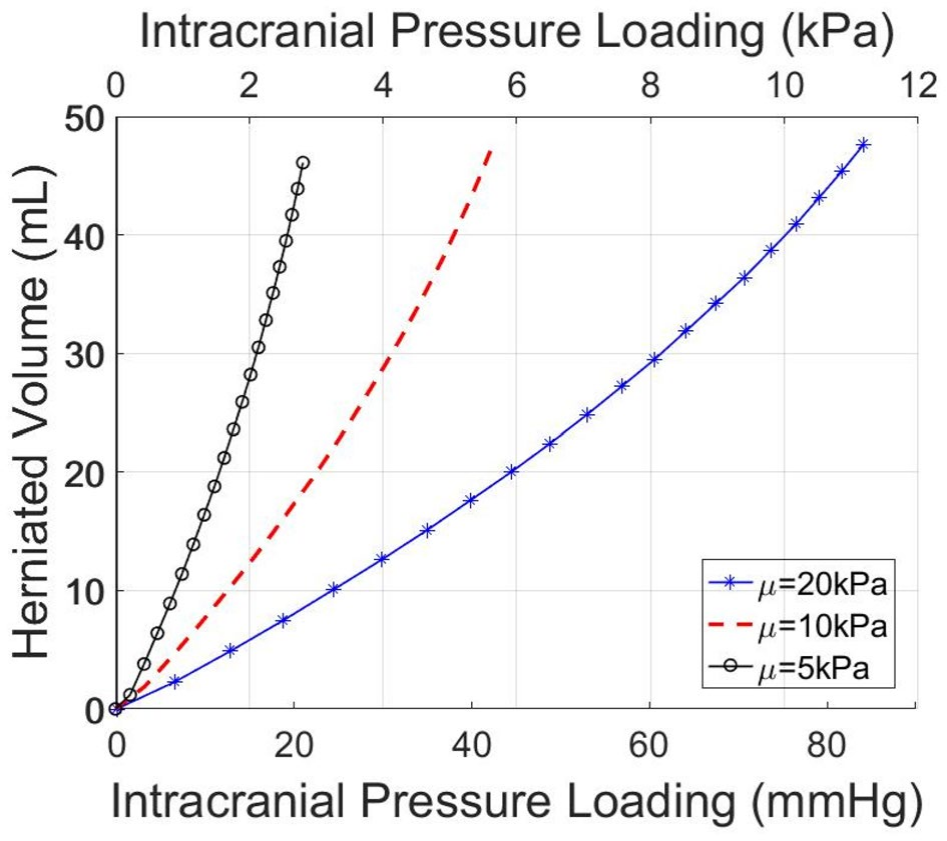
Brain herniated volume versus ICP loading for a circular unilateral craniectomy—dependence with respect to shear modulus, μ**.** In silico predicted herniated volume of the brain tissue following craniectomy as a function of the intracranial pressure loading prior to DCC for the three values of the shear modulus, $\mu$, considered.

Figure 8 illustrates the intracranial pressure prior to the decompressive craniectomy procedure in relation to the predicted herniated volume following craniectomy for the three values of shear modulus considered. The stiffer the brain tissue, the less volume of it will herniate for the same intracranial pressure levels. According to a clinical study performed on 21 patients with a mean age of 39.4 years^23^, the mean ICP has been reported at 36.4 mm-Hg (ICP measured range: 18–80 mm-Hg) while the herniated volume of the brain tissue was 98 ml where on average a unilateral craniectomy of 8800 mm^2^ was considered. However, assuming here that the physiological ICP value of an adult at rest is 10 mm-Hg, the results with respect to the intracranial pressure loading will need to be incremented by 10 mm-Hg to give the exact ICP value prior to DCC. Thus, the model predictions of a unilateral craniectomy of 5026 mm^2^ (r = 40 mm) agree well against the clinical evidence for when a 5–10 kPa shear modulus is adopted in the brain biomechanical model (see paragraph: Linear momentum balance). On the contrary, the in silico results for a shear modulus of 20 kPa (or above) do not agree with the clinically reported herniated volume measurements—a stiffer brain model gives lower tissue deformation predictions.

## Discussion

In this study, we present a FE model of post-traumatic brain injury and decompressive craniectomy that incorporates a biphasic, nonlinear biomechanical model of the brain. We used the model to perform a series of simulations to investigate the effect of the opening of craniectomy on the herniated volume of the brain tissues as a function of the ICP. We further investigated the stresses and deformations within the brain during craniectomy, the effect of the brain mechanical properties on the tissue deformation and the displacement of the shift of the brain’s midline. Our result for the magnitude of the stresses and deformations agree with previous modelling studies but also with clinical measurements. The range of ICP in clinical measurements has been reported in the range 18–80 mm-Hg with a mean value 36.4 mm-Hg approximately^23^, while the herniated volume of the brain tissue ranged 27–127 ml^24^. For the same clinical scenarios of DCC, the proposed biomechanical model gives ICP and herniated volume predictions that agree very well with the clinical evidence. Also, our model can produce realistic simulation outputs of the brain tissue deformation (including mid-line shift) after craniectomy surgery^27^. In addition, our model predictions agree qualitatively with previous simulation studies in such that the injury and the opening should be on the same side of the brain so that the brain midline shift is minimal^8^. However, in this study, we put additional emphasis on the relationship between the herniated volume and the size of craniectomy opening (see also plots of herniated volume versus size in Fig. 3 in Appendix B). The herniated volume is a key parameter in DCC surgery. Indeed, clinical studies have reported measurements of the herniated volume^23,24^ to define the appropriate size of the craniectomy opening (area), while others attempt to derive relationships using demographic data, herniation volume, and the maximum extracranial brain herniation distance^27^. Equally important to this, clinical evidence suggests that infection and complications of wound healing are strongly related to the opening size and, thus, the herniated volume of the brain tissue after DCC^8^. The model highlights the strong effect of the size of the openings and the mechanical properties of the brain tissue on the herniated volume (Figs. 3, 5 and 8). Importantly, small variations in the mechanical properties of the brain can swift considerably the herniated volume versus ICP curve (Fig. 8). Non-invasive methods, such as magnetic resonance elastography, could provide more accurate assessment of the mechanical properties of the brain and reinforce the validity of the model towards producing patient-specific predictions.

Our model is limited in that it models the solid phase of the brain as an isotropic neo-Hookean nonlinear elastic material and assumes the same shear and bulk modulus throughout the tissue. The isotropic Mooney-Rivlin or the simple Ogden constitutive equations have also been employed in pertinent studies^8,9,28^, which is still considered a limitation as the brain tissue can exhibit a non-linear biomechanical response and the tissue may not behave isotropically. Also, given the large variability of data for the mechanical properties of the different compartments of the brain found in the literature^9,17,21^, incorporating the grey and white matter as two different materials would still involve uncertainties that limit the model predictions. However, elastography imaging could be considered to providing accurate estimate of the tissue elasticities and their distribution within the entire brain and, therefore, reduce these uncertainties. Furthermore, our model does not account for other structural components of the brain, such as the dura matter, the falx and tentorium. How incorporation of these components would affect our simulations is not intuitive and detailed simulations would have to be performed. Additionally, TBI usually causes swelling in a specific area of the brain, however, we assumed that the whole brain was considered injured (swollen) expect in the case of decompressive craniectomy at injured and non-injured side, where only one hemisphere of the brain was considered injured. Finally, we modeled only for the equilibrium state of the brain following craniectomy and, hence, the model cannot predict the transition of ICP after opening of the brain.

To conclude, we developed a computational framework for the study of DCC focusing on the herniated volume as a function of the size and shape of the opening and the ICP. We also to notice that the model predicts ICP loading, and this means that the ICP loading will need to be added to physiological ICP (i.e. between 7 and 15 mm-Hg in adults at rest) to provide the clinical ICP value prior to DCC. In practice, physicians can measure the ICP on patients with traumatic brain injury and then calculate the difference between this pressure and the physiological one. Therefore, physicians will be able to use our graphs based on this pressure (i.e. ICP loading) and predict the herniated volume, stresses and deformations within the brain during craniectomy.

## Supplementary information

Supplementary Information

## Data Availability

The data that support the findings of this study are openly available on Figshare. More specifically, for the unilateral craniectomy simulations (Figs. 2 and 3), the FE model data are available in 10.6084/m9.figshare.12546428, while for the bifrontal craniectomy simulations (Figs. 4 and 5) the model data are available at 10.6084/m9.figshare.12546386. The model data for the swelling comparison study on the unilateral craniectomy scenario (Fig. 6) they can be found at 10.6084/m9.figshare.12546473. Also, for the unilateral craniectomy simulations of the shear modulus comparison (Figs. 7 and 8) the FE model data are available at 10.6084/m9.figshare.12546464.
