# Supplementary material for: Decompressive craniectomy of post-traumatic brain injury: an in silico modelling approach for intracranial hypertension management
Source: Sci Rep. 2020 Oct 29;10:18673. doi: 10.1038/s41598-020-75479-7 (PMC7596483; doi:10.1038/s41598-020-75479-7)
Supplement: Supplementary file 1 — Supplementary Information [file 41598_2020_75479_MOESM1_ESM.docx]

**Supplementary Information**

**Decompressive craniectomy of post-traumatic brain injury: an in silico modelling approach for intracranial hypertension management**

**C. Lambride, N. Christodoulou, A. Michail, V. Vavourakis, T. Stylianopoulos**

# **Appendix A**

To ensure the accuracy and the stability of the COMSOL simulation results, a mesh convergence study was performed. Three different FE mesh densities were considered for the unilateral craniectomy model (with an opening radius 20 mm): a coarse density mesh consisted of 164,926 tetrahedral finite elements and 34,958 nodes, a moderately dense mesh consisted of 277,364 tetrahedral finite elements and 57,879 nodes, and a denser mesh consisted of 2,470,365 elements and 481,367 nodes. To assess the mesh convergence, the numerically predicted solid stresses and maximum displacement values were compared. Assuming the denser mesh (due to the extra fine spatial resolution) produces a solution very close to the exact one, the predictions of the moderately dense mesh compared (against the denser mesh) by approximately 1% and 3% in terms of the maximum displacement and solid stresses respectively, while for the coarse density mesh they compared by approximately 4% and 6% in terms of the maximum displacement and solid stresses respectively. Thus, and in view of the significant computational burden to run simulations for the dense meshes (coarse mesh run in about 12 hours on a desktop machine while the dense mesh run for one week approximately), the coarse mesh was considered acceptable for the present FE analysis.

# **Appendix B**

$$\boldsymbol{u} = \boldsymbol{0}$$

$$\boldsymbol{n\cdot}\mathbf{v}^{\boldsymbol{f}}\boldsymbol{=}0$$

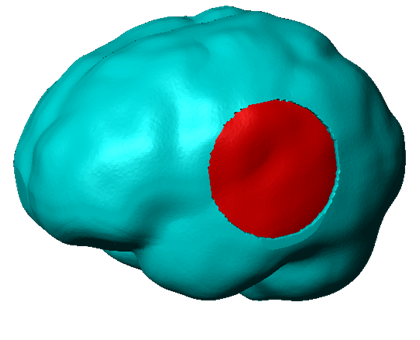

$$\boldsymbol{n\cdot}\boldsymbol{\sigma}^{\boldsymbol{s}}\boldsymbol{=}0$$

$$p_{i} = 0$$

**Figure 1: Boundary conditions of the 3D model.** The skull was modeled as a rigid body (i.e., $\boldsymbol{u} = \boldsymbol{0}$) and a zero-flux boundary condition ($\boldsymbol{n\cdot}\mathbf{v}^{\boldsymbol{f}}\boldsymbol{=}0$) was applied for the fluid phase at the skull/brain interface, where $\boldsymbol{n}$ corresponds to the outward unit normal vector to the surface of the 3D model. The brain tissue at the skull opening was unconstrained (i.e., traction-free: $\boldsymbol{n\cdot}\boldsymbol{\sigma}^{\boldsymbol{s}}\boldsymbol{=}0$), while the fluid pressure at the opening was zero ($p_{i}=0$).


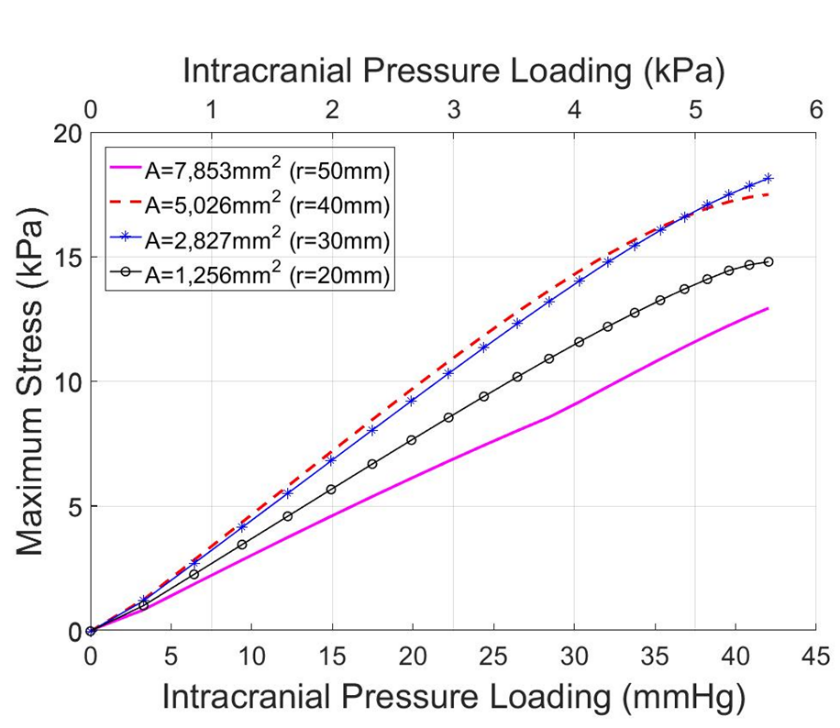


**A**


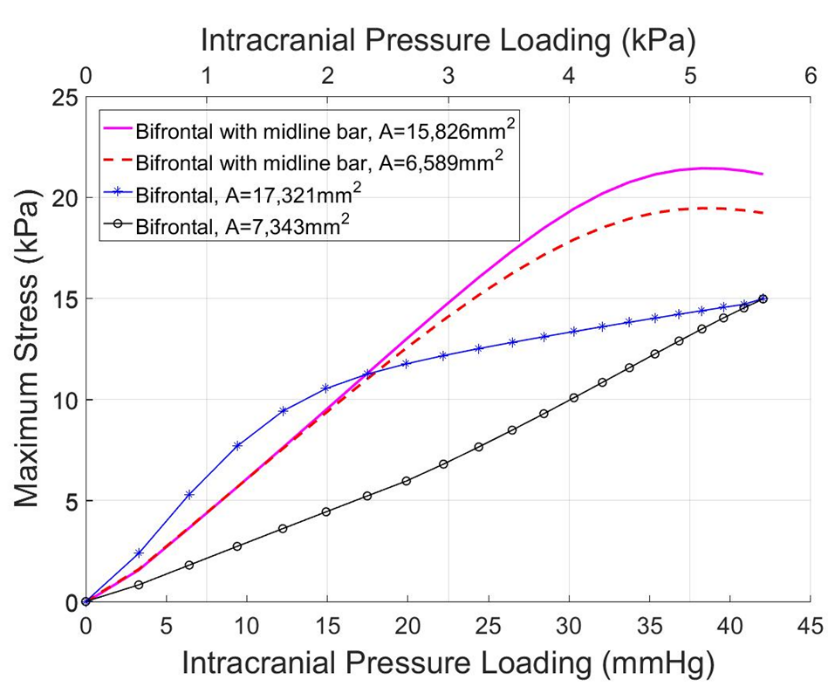


**B**

**Figure 2: Line plots of brain maximum stress versus ICP loading for circular unilateral and bifrontal craniectomy.** In silico-predicted maximum stress of the brain tissue following craniectomy for different (A) circular and (B) bifrontal (with or without midline bar) craniectomy openings as a function of the intracranial pressure loading prior to DCC.


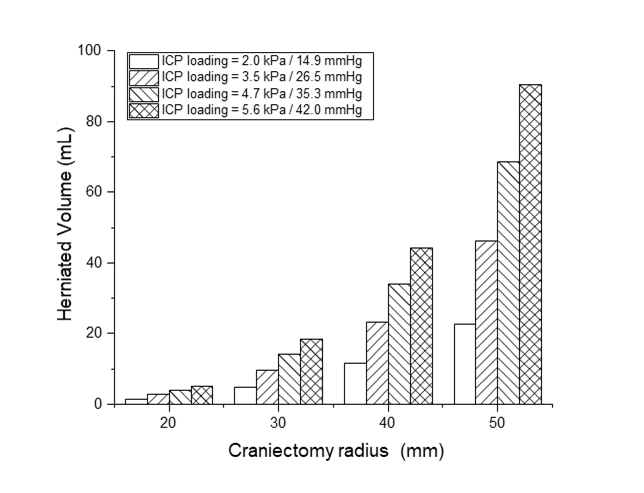

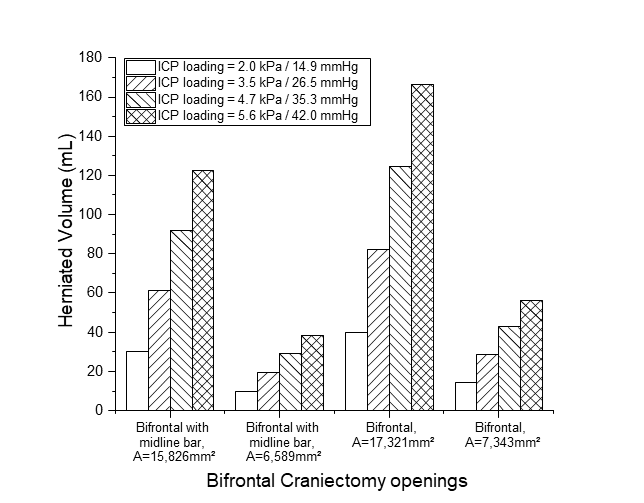


**A**

**B**

**Figure 3: Bar charts of brain herniated volume versus craniectomy opening size under varying ICP loadings.** In silico-predicted herniated volume of the brain tissue following craniectomy for different geometry openings under varying intracranial pressure loading prior to DCC.


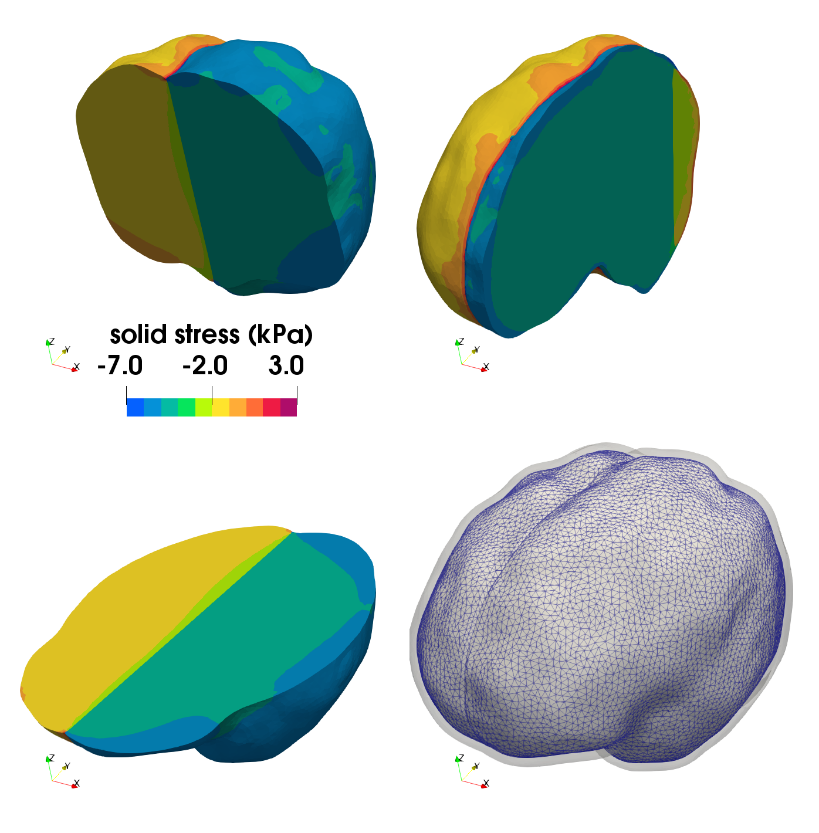


**Figure 4: Stressed developed before DCC for a one hemisphere injury scenario.** Spatial distribution of the brain tissue solid stresses (in kPa) for a half-brain injury and prior to DCC: scenario considered for a 10% tissue swelling on the right hemisphere.
